# Study on temporal and spatial differentiation of biocapacity in Shenyang from a multi-scale perspective

**DOI:** 10.1371/journal.pone.0263601

**Published:** 2022-02-07

**Authors:** Yanpeng Gao, Wenjun Chen, Chunyao Guo

**Affiliations:** Jangho Architecture College, Northeastern University, Shenyang, Liaoning Province, China; United Nations University Institute for Natural Resources in Africa, GHANA

## Abstract

Biocapacity of a region exhibits spatial differences owing to the limitations of regional scale and natural conditions. Based on the multi-scale perspective, we comprehensively studied and analyzed the temporal and spatial differences of the biocapacity of a region in an attempt to establish the groundwork for optimizing urban development and its utilization framework. By adopting the ecological footprint model along with multi-scale difference evaluation method, the municipal and county scales are incorporated into a unified analysis framework in this paper, thereby facilitating the exploration of the temporal and spatial differences in the biocapacity of Shenyang—a city in China—from 2005 to 2019. The results demonstrated that: 1) At the municipal scale, the biocapacity per capita fluctuated between 1.35 hm^2^/person and 2.22 hm^2^/person. It revealed an “up-down-up” trend, which appeared consistent with the Kuznets cycle; at the county scale, the biocapacity depicted spatial differences, while those of downtown and surrounding districts/counties developed a two-level ascending hierarchical structure. 2) The time series of footprint size and depth first ascended and then declined, and can be classified into four types: closed type, inverted U-type, S-type, and M-type. Among them, S-type and M-type have the phenomenon of over-utilizing the stock capital. 3) For a long time, the regional difference of biocapacity has mostly dwelt on two scales with an evident scale effect, and the biocapacity of Liaozhong District was the worst.

## 1. Introduction

Since the reform and opening-up campaign in China, the urbanization process has witnessed rapid advancement. With an excessive occupation of vast proportions of agricultural and ecological land, the conflict between urban economic and social development as well as ecological environment continues to intensify, along with the ever more apparent resource constraints. Hence, it is imperative to systemize and optimize the relationship between urban construction and the natural system [1,2]. In this context, the concept of biocapacity was proposed and has been gradually evolving in the field of urban planning as an imperative index for evaluating the regulation capacity, maintenance capacity, and the support capacity of the urban-natural system. Owing to the distinctive regional characteristics of the natural resource distribution in China, the economic and social development as well as the natural resource utilization efficiency tends to vary in different regions. Hence, exploring the temporal and spatial differentiation characteristics of urban biocapacity appertaining to the regional resource endowment can be highly conducive for promoting urban high-quality development, sustainable construction, as well as sustainable utilization of the available resources.

In recent years, both Chinese and foreign scholars have conducted comprehensive research on biocapacity, majority of which emphasized on connotation evaluation [3], evaluation index [4], evaluation practices [5], etc. Methods such as state-space method [6], comprehensive evaluation method [7,8], and energy analysis method [9] were primarily adopted for connotation evaluation, to concentrate on the comprehensive evaluation of the concept and connotation of biocapacity; evaluation indexes were largely employed for the DPSIR conceptual model [10], PSR conceptual model [11,12], ecological footprint model [13], etc., to create evaluation indexes concerning biocapacity from different dimensions, and evaluation models such as principal component analysis model [14], grey system model [15], multiple regression model [16], etc., were employed to achieve quantitative analysis. With respect to evaluation practices, the CA Markov model, neural network model, OLS model, GWR Model, etc., [17–19] were often implemented for evaluating urban biocapacity, as well as for assessing aspects like influencing factors, capacity improvement, prediction, early warning, etc. In a nutshell, evaluation studies on biocapacity have been getting relatively mature, but majority of the existing studies are centered on the large and medium scales such as watershed, urban agglomerations, and provinces, although small-scale studies at county level are involved, they are mostly limited to a single level at city or county, and comparative analysis of multi-scale biocapacity is relatively rare. In the rapid progress of urbanization,due to the difference in the environmental background and the ability to obtain resources in urban space, county economic development is more urgent, land use is more rough, economic development relies more on natural capital, and there is a significant difference in time and space from the urban area.Therefore, multi-scale comprehensive control of the differentiation characteristics of biocapacity is an important basis to optimize urban ecological pattern.

With most of the northeastern Chinese cities successively entering the mature or declining period of resource development in recent years, Shenyang as the central northeastern city and the chief growth pole for the invigoration of the old industrial base has also been challenged with the realistic dilemma of constrained resources and intensified conflict between urban construction and resource and environment. Therefore, a comprehensive understanding of the temporal and spatial differentiation characteristics of urban biocapacity is instrumental for enhancing the quality of urban development, development coordination, and pattern protection. Against this background with Shenyang as an example, we review the temporal and spatial differentiation characteristics of urban biocapacity at the municipal and county scale level in this paper by adopting the ecological footprint temporal and spatial difference model and multi-scale difference evaluation method. And identify the type and extent of multi-scale changes in biocapacity, and analyze natural capital occupation and regional differences. It integrates the logical framework of "conceptual cognition-spatiotemporal evolution-influencing factors" in the time dimension and "type division-difference analysis-protection confirmation" in the spatial dimension. This study identifies urban multi-scale ecological carrying capacity from the perspective of time and space, and aims to provide a reference for the analysis of ecological carrying capacity of megacities in the process of rapid urbanization. At the same time, it has guiding significance for promoting the optimization of urban ecological pattern.

## 2. Data sources and methodology

### 2.1 Overview of the study area

**Fig 1:**
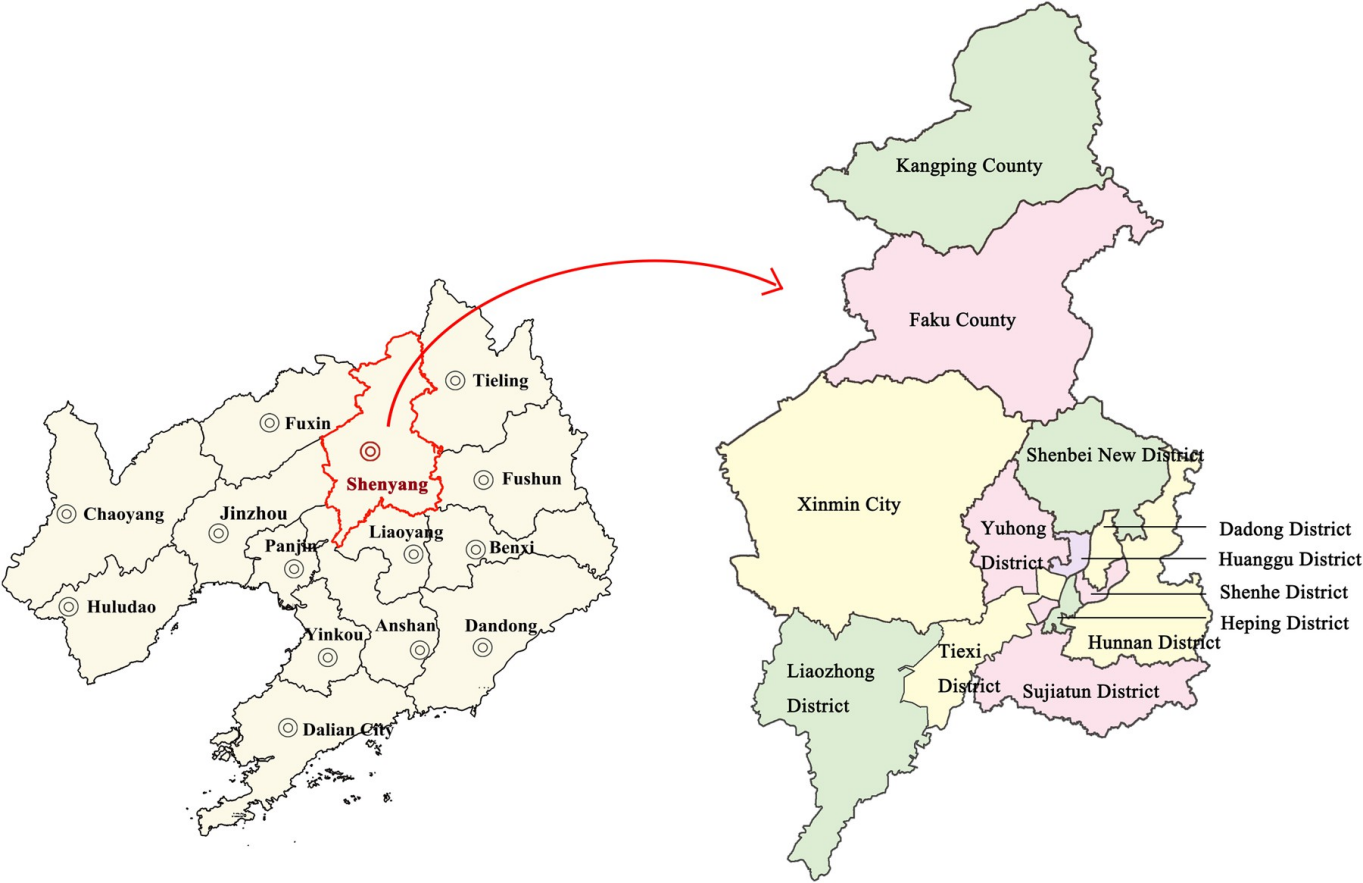
Study area (Image source: https://www.naturalearthdata.com/downloads/10m-cultural-vectors/).

Shenyang city (Fig 1) is located in the south region of northeast China and the central northern part of Liaoning Province, ranging in the latitude from 41°11′51″– 43°2′13″ and the longitude from 122°25′9″– 123°48′24″. It comprises of 10 municipal districts, 2 counties, and 1 county-level city (under temporary administration), covering a total land area of 12,948 km^2^. Shenyang is located in the central area of Liaohe plain, surrounded by picturesque hills and mountains on its east and north regions. Its terrain gradually flattens out towards the west and south. Shenyang serves as the political and cultural capital of northeast China. Positioned in the middle of Northeast Asia Economic Circle and Bohai Economic Circle, Shenyang happens to be a mega city in northeastern China. Nevertheless, the constant development of economy and society in recent years has resulted in the increasingly severe conflict between human activities and natural environment, which proves detrimental for upholding the premium sustainable development of the city.

### 2.2 Data sources

Geospatial data are selected from Landsat4-5 TM and Landsat 8 OLI_TIRS satellite images and ASTER GDEM elevation data from 2005 to 2019, with a spatial resolution of 30 m, data are obtained from geospatial Data Cloud Platform of Computer Network Information Center, Chinese Academy of Sciences (http://www.gscloud.cn). The remote sensing images of Shenyang were interpreted by ENVI5.0, the images were preprocessed by geometric correction and image enhancement, and supervised classification and manual visual interpretation were used, and administrative boundary of Shenyang derived from the geographic information service platform of Liaoning Province (http://liaoning.tianditu.gov.cn/) was superimposed on ArcGIS16.0, finally the land use status data of Shenyang from 2005 to 2019 were obtained.

Social statistics include 2006–2020 *Shenyang City Statistical Yearbook*, *Shenyang City National Economic and Social Development Statistical Bulletin*, *Shenyang City Rural Statistical Yearbook*, *Shenyang City District and County Statistical Yearbook* and *China Agricultural Statistics*, the data comes from Shenyang Statistics Bureau (http://tjj.shenyang.gov.cn/), the People’s Government of Liaoning Province (http://www.ln.gov.cn/), statistical yearbook sharing platform (https://www.yearbookchina.com/) and cnki.net (https://data.cnki.net/). In addition, the equilibrium and yield factors of the diverse types of land areas are taken from the Working Guidebook to the National Footprint and Biocapacity Accounts published by Global Footprint Network (https://www.footprintnetwork.org/)in 2019.

### 2.3 Methodology

#### 2.3.1 Ecological footprint temporal and spatial difference model

1) Ecological footprint depicts an estimate of ecosystem pressure, and particularly refers to the sum of the total resources consumed for steadily sustaining the population and the bioproductive land required for accommodating the waste generated under a certain economic environment [20]. The calculation formula is as follows:

$$EF=N\times ef=N\times \sum_{j=1}^{n}r_{j}\times A_{i}=N\times \sum_{j=1}^{n}r_{j}\times (c_{i}/p_{i})$$ (1)

Where, *EF* is the total amount of regional ecological footprint; *N* is the population of the region; *ef* represents the ecological footprint per capita; *A*_*i*_ refers to the bioproductive land area per capita converted for the consumption item i; *c*_*i*_ is the consumption per capita of item i; *p*_*i*_ depicts the average production capacity of consumption item i; *and r*_*j*_ is the equilibrium factor.

On account of the limitations presented by various statistical capacities in different regions and inadequate continuum of the resources data, the accounting of ecological footprint in this study only includes the data of biological resources, which is composed of 10 items such as agricultural products, forest products, livestock products, aquatic products, etc. All the equilibrium and yield factors of the diverse types of land areas adopted in this paper have been taken from the *Working Guidebook to the National Footprint and Biocapacity Accounts* published by Global Footprint Network in 2019 [20].

2) Biocapacity denotes the sum of bioproductive land areas that can be utilized for human needs in a certain area, and its calculation formula is as follows:

$$EC=N\times ec=N\times \sum_{J=1}^{N}A_{j}\times r_{j}\times y_{j}$$ (2)

Where, EC is biocapacity; *N* refers to the regional population; *ec* represents biocapacity per capita; *A*_*j*_ refers to biological production area per capita; *r*_*j*_ and *y*_*j*_ represent the equilibrium factor and yield factor of land type j, respectively.

3) Ecological balance signifies the difference between ecological footprint and biocapacity, and its calculation formula is as follows:

ED=EC−EF (3)

Where, ED is the ecological deficit (surplus). If ED>0, the regional ecology tends to be in the surplus state, and if ED<0, the regional ecology is in the deficit state.

4) The three-dimensional ecological footprint model [21], which is based on the two-dimensional footprint model, is employed by Niccolucci and several other scholars for distinguishing between flow capital and stock capital, and further exemplifying the occupation of flow capital and stock capital in accordance to the footprint size and depth. The calculation formula is as follows:

$${EF}_{depth}=1+\frac{ED}{EC}=\frac{\sum max({EF}_{i}-{EC}_{i},0)}{EC}$$ (4)

$${EF}_{size}=\frac{EF}{{EF}_{depth}}$$ (5)

Where, *EF*_*depth*_ represents the depth of ecological footprint, *EF*_*size*_ denotes the size of ecological footprint, *EF*_*i*_ is the ecological footprint of Class I land, *and EC*_*i*_ represents the biocapacity of Class I land. If and only if EF≤EC, the *EF*_*depth*_ is 1, and flow capital can fulfill the need of consumption; if EF > EC, then *EF*_*depth*_>1, which symbolizes capital loss, and indicates that the flow capital cannot fulfill the need of consumption, and the stock capital needs to be utilized.

#### 2.3.2 Grey relational analysis

Gray relational analysis is a method to measure the degree of correlation between factors based on the same or different development trends of historical data sequences [22], we use this method to calculate the relevance between biocapacity and its influencing factors. The calculation formula is as follows:

$$\bar{S}=\frac{1}{n}\times \sum_{t=1}^{n}S(t)$$ (6)

Where, S(t)is the correlation coefficient between the influencing factors of the comparison sequence and the reference sequence in year t.The calculation formula is as follows:

$$S(t)=\frac{\Delta_{min}+\rho \times \Delta_{max}}{\Delta (t)+\rho \times \Delta_{max}}$$ (7)

Where: Δmin and Δmax are the minimum and maximum values of the absolute difference between the comparison and the reference sequence in year t; Δ(t) is the absolute difference between the comparison and the reference sequence in year t; ρ is the distinguishing coefficien, generally between 0–1, usually 0.5.

#### 2.3.3 Multi-scale difference evaluation method

1) Difference index measurement model. This paper uses the city and county levels as the research scale, so that the difference index measurement model is selected to measure the standard deviation and coefficient of variation between and within regions. In the case of clear expression of differences, the research method is more straightforward.Aiming to meticulously reflect the multi-scale characteristics of biocapacity in Shenyang, two methods—absolute difference and relative difference—were employed to quantitatively evaluate the multi-scale biocapacity difference. Standard deviation was used for obtaining absolute difference; while the variation coefficient was utilized for relative difference [23]. The calculation formula is as follows:

$$S=\sqrt{\frac{1}{N}\sum_{i=1}^{N}(}x_{i}-{\bar{X})}^{2}$$ (8)

$$C_{v}=\frac{S}{\bar{X}}$$ (9)

Where, *S* and C_*v*_ represent the standard deviation and variation coefficient of ecological footprint per capita and biocapacity per capita of a certain scale, respectively; i represents the research unit of a certain scale (municipal level, district (county) level); *x*_*i*_ denotes the ecological footprint per capita and biocapacity per capita of unit i; $\bar{X}$ is the simple arithmetic mean value of the ecological footprint per capita and biocapacity per capita of all the units in a certain scale; N is the total number of research units within a certain scale. The greater the S value, greater is the absolute difference of regional biocapacity, and vice versa. The greater the value of C_*v*_, greater is the relative difference of biocapacity, and vice versa.

2) Relative development model. To identify the gap between the response intensity of biocapacity of every county and the whole region of Shenyang during different time periods, the relative development model is introduced [24]. The calculation formula is as follows:

$$N=\frac{\left(X_{Ti}-X_{ti}\right)}{\left(\bar{X_{T}}-\bar{X_{t}}\right)}$$ (10)

Where, *X*_*Ti*_ and *X*_*ti*_ represent the response value of ecological footprint per capita and biocapacity per capita of county i at the time of t and T, respectively. $\bar{X_{T}}\;and\;\bar{X_{t}}$ symbolize the mean value of ecological footprint per capita and biocapacity per capita at the time of t and T, respectively. The greater the N value, greater is the gap between the biocapacity of every district/county and Shenyang during different periods, and vice versa.

3) Dispersion method. Aiming to manifest the general impact of the response value of ecological footprint per capita and biocapacity per capita of every county on the average level of the whole region, the dispersion method is introduced [25]. The specific formula is as follows:

$$D_{i}=X_{i}-\bar{X}$$ (11)

Where, *X*_*i*_ denotes the response value of ecological footprint per capita and biocapacity per capita of county i, and $\bar{X}$ represents the average value of the whole region. The greater the *D*_*i*_ value, greater is the biocapacity of every district/county relative to the average level of Shenyang, and vice versa.

4) Theil index. Theil index was first proposed by Henri Theil in 1967. It is a scientific index that measures the inequality or the difference between a group of data. The closer the value is to 0, the smaller the difference is between the regions; the larger the value, the greater is the difference between the regions [26]. Theil index is an important method to measure the internal differences of various regions. In this study, the Theil index method was adopted to analyze the internal difference of biocapacities within Shenyang. The calculation formula is as follows:

$$T=\sum_{i=1}^{n}tef_{i}ln\frac{tef_{i}}{tec_{i}}$$ (12)

Where, n is the number of districts (counties), and *tef*_*i*_ denotes the ecological footprint per capita of district/county i; *and tec*_*i*_ is the biocapacity per capita of district/county i. The smaller the value of T, smaller is the difference of biocapacity among districts/counties, and vice versa.

## 3. Results

### 3.1 Analysis of biocapacity evolution

#### 3.1.1 Municipal scale

**Fig 2:**
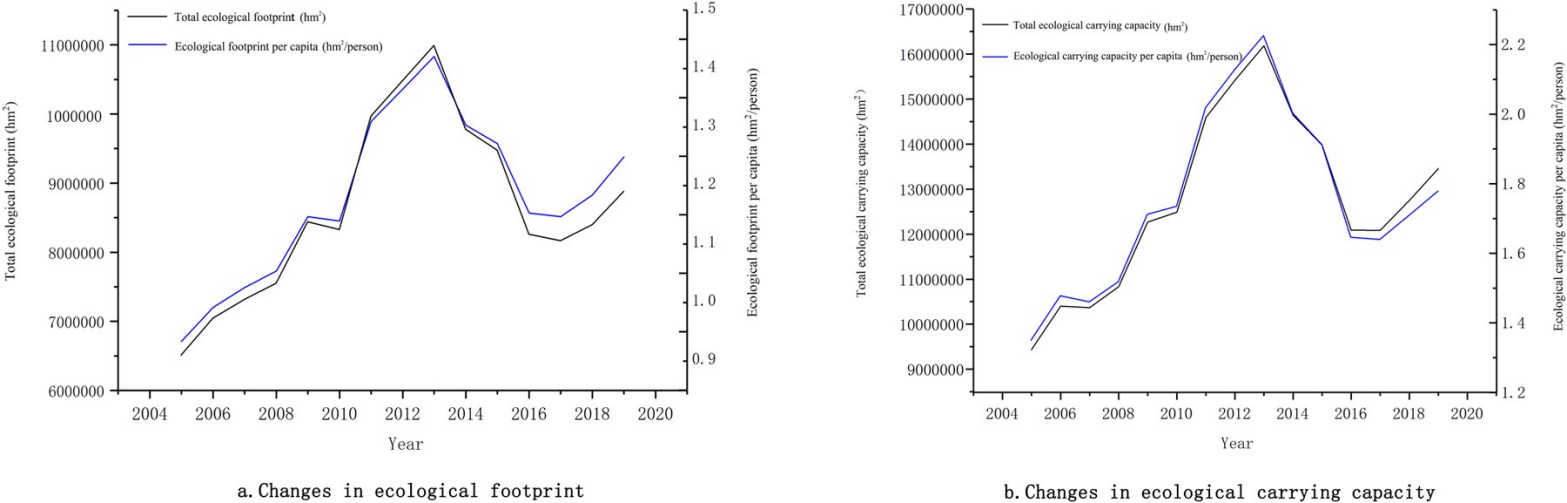
Trends in ecological footprint and ecological carrying capacity of Shenyang City, 2005–2019. a. Changes in ecological footprint. b. Changes in ecological carrying capacity.

The measurement of ecological footprint of Shenyang from 2005 to 2019 reveals that it illustrates a fluctuating up-down-up trend (Fig 2A), which appears to be consistent with the fluctuation of Kuznets cycle. In particular, from 2005 to 2013, the total ecological footprint showed a massive surge, from 6,707,779square kilometer(hm^2^) to 10,831,038 hm^2^, and the ecological footprint per capita increased from 0.96 hm^2^/person to 1.49 hm^2^/person, attaining its peak in 2013. This surge could mainly be attributed to two reasons. On one hand, with the unceasing development of economy and society, the demand for natural resources during urban development grew constantly, which led to the rapid expansion of the productive area. On the other hand, the continuous advancement of the living standards of people led to the surge of consumption of items derived from biological resources, thus resulting in the rapid consumption of ecological resources in the city. From 2013 to 2017, the total ecological footprint decreased from 10,831,038 hm^2^ to 8,515,772 hm^2^, and the ecological footprint per capita decreased from 1.49 hm^2^/person to 1.16 hm^2^/person. Both of these indicators illustrated a downward trend. This signifies that during that period, Shenyang possibly devoted more attention to urban ecological security, which caused the decrease of the ecological footprint. From 2017 to 2019, the above-mentioned two indicators experienced an upward trend again, with an increase of 864,443 hm^2^ and 0.08 hm^2^/person, respectively. During that period, the economic structure in Shenyang had been altered, and eco-tourism, such as characteristic fruits picking and agricultural sightseeing, was vigorously developed. Subsequently, the consumption of natural resources witnessed a substantial surge again in the production and life of people.

The biocapacity of Shenyang demonstrated a fluctuating up-down-up trend (Fig 2B), and its fluctuation size seemed bigger than that of its ecological footprint. It exhibited a constant increase from 2005 to 2013 and attained its peak in 2013. The total biocapacity and biocapacity per capita were recorded to be 16,181,958.62 hm^2^ and 2.23 hm^2^/person, respectively. From 2013 to 2017, the indicators decreased to 12,085,976.11 hm^2^ and 1.64 hm^2^/person; after 2017, they experienced an upward trend again. By and large, Shenyang’s biocapacity synchronized well with the adjustment in its ecological footprint, and was considerably influenced by this shift, which indicates that during the process of urban development, the constant adjustment of urban land utilization can produce extensive effects on the required ecological and productive land, thus leading to dynamic changes of biocapacity.

#### 3.1.2 County scale

**Fig 3:**
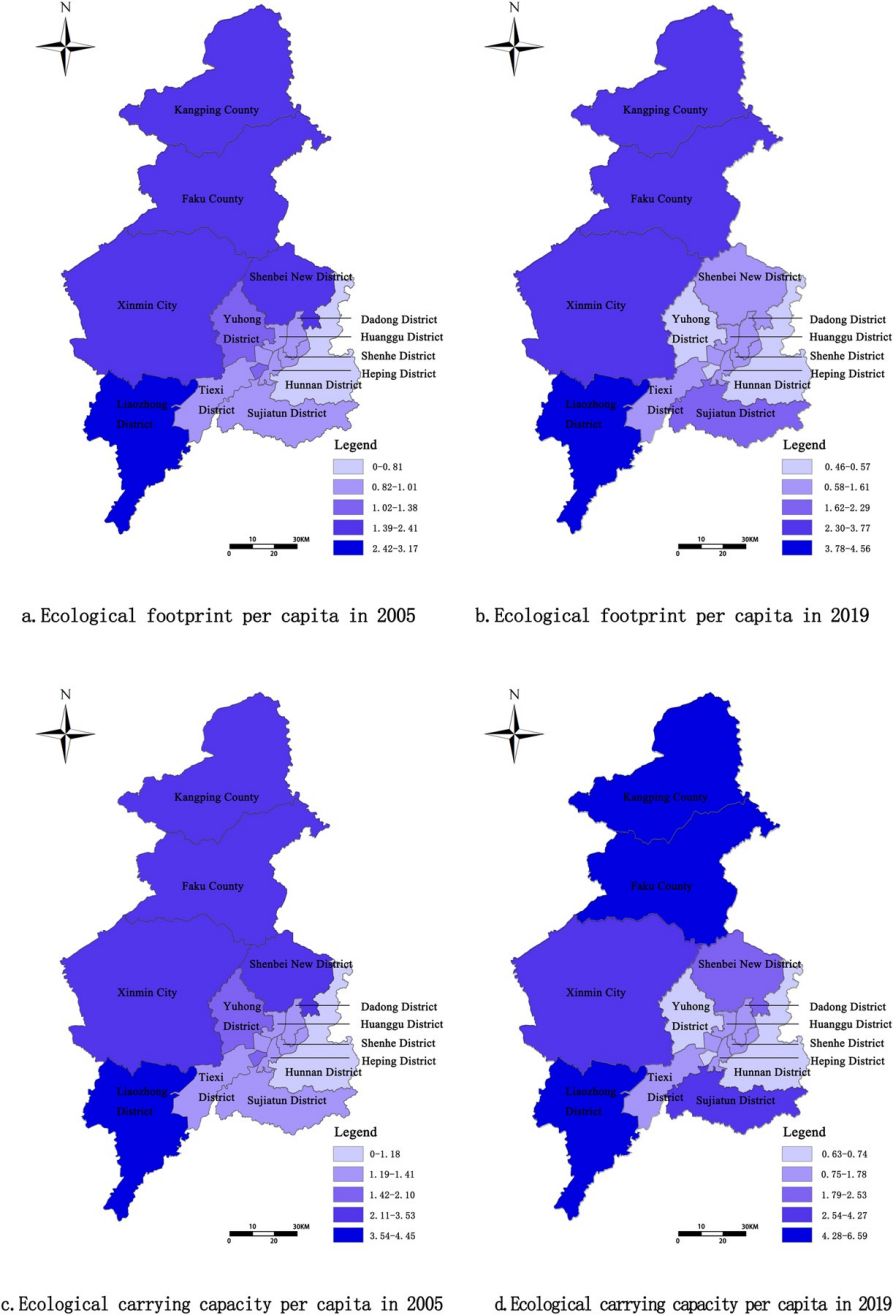
Spatial evolution pattern of ecological footprint and ecological carrying capacity of Shenyang in 2005 and 2019 (Image source: https://www.naturalearthdata.com/downloads/10m-cultural-vectors/). a. Ecological footprint per capita in 2005. b. Ecological footprint per capita in 2019. c. Ecological carrying capacity per capita in 2005. d. Ecological carrying capacity per capita in 2019.

Experiencing different paces of economic development can lead to significant spatial differences in the ecological footprint of an area (Fig 3A and 3B). In terms of biocapacity from 2005 to 2019, downtown and surrounding districts/counties of Shenyang developed a two-level (from low to high) hierarchical structure in space. In particular, in 2005, Liaozhong District (3.17 hm^2^/person), Kangping County (2.41 hm^2^/person), Faku County (2.08 hm^2^/person), Shenbei New District (2.01 hm^2^/person), and Xinmin City (1.97 hm^2^/person) were the counties with the highest ecological footprint per capita. Traditional agriculture was the chief form of economic structure in most of these areas. With little progress in the domain of economic structure, the economic construction of these areas was highly dependent on the ecologically productive land such as cultivated land, forest land, and grassland, leading to a high ecological footprint per capita. In 2019, the ecological footprint per capita of Shenbei New District and Yuhong District decreased from 2.01 hm^2^/person to 1.61 hm^2^/person and 1.38 hm^2^/person to 0.57 hm^2^/person, respectively. This decrease could mainly be attributed to the gradual implementation of regional development planning, the optimization and regulation of industrial structure, and the more rationalized and effective utilization of the available natural resources.

The biocapacity per capita exhibited considerable spatial differences in Shenyang (Fig 3C and 3D), portraying a spatial pattern of low in downtown areas and high in suburban areas, which appeared consistent with the levels of regional economic development. In 2005, Liaozhong District had the highest biocapacity per capita, which was 4.45 hm^2^/person, and Hunnan District maintained the lowest biocapacity per capita, which was 1.18 hm^2^/person. In 2019, Faku County experienced the fastest rise of biocapacity per capita, with an increase of 2.14 hm^2^/person, and Hunnan District witnessed the fastest decrease of 0.55 hm^2^/person. Such variations were taking place due to the emanating leading effect of Shenyang’s rapid economic development. The socio-economic activities of surrounding counties (incl. Faku County) was gradually progressing as well, leading to the intensification of land development and utilization, which in turn also added to the demand for land resources and ecological environment, burdening the regional biocapacity further beyond its tolerable level.

#### 3.1.3 Influencing factors

The rapid advancement of urbanization has caused profound changes in the natural and social systems of the city. The continuous development of economy and society has provided a necessary foundation for the upgrading of the city. However, disorderly urban construction consumes a large amount of natural resources and severely squeezes the natural space, the urban ecosystem is facing severe challenges. With the increase of urban population, the relationship between man and land is driven by more complex interests, and the structure and function of urban space change dramatically. At the same time, in order to meet the needs of regional development and residents’ living during the development process, urban space has continuously adjusted the urban land utilization, which affects changes in ecological and productive land area and leds to dynamic changes of biocapacity.

Based on this, this paper selects seven indicators of GDP, GDP per capita, population size, population density, urbanization rate, ecological land ratio and ecological area per capita, and uses gray relational analysis (formula 6) to explore the main factors in the change of biocapacity in Shenyang.

From 2005 to 2019, the biocapacity of Shenyang and the influencing factors selected showed different correlation sequences (Table 1). Among them, the ecological footprint of Shenyang is most affected by ecological area per capita, with the degree of association of 0.6545, and is less affected by GDP, with the degree of association of 0.4632. The biocapacity is most affected by ecological area per capita, with the degree of association of 0.6646, and less affected by GDP, with a correlation degree of 0.4833. It can be seen that the ecological footprint and biocapacity of Shenyang is significantly affected by ecological area per capita, while the direct effect of economic development on biocapacity is not obvious. The reason is that due to the emanating leading effect of Shenyang’s rapid economic development, the socio-economic activities of surrounding counties (incl. Faku County) was gradually progressing as well. The demand for land resources and ecological environment has increased, natural ecological land space has been severely squeezed in the process of urbanization, ecological area per capita has been significantly reduced, the regional ecological carrying burden is increasing, and the urban ecological carrying capacity changes obviously.

**Table 1:** Correlation between biocapacity and influencing factors in Shenyang from 2005 to 2019.

Biocapacity	GDP	GDP per capita	population	population density	urbanization rate	ecological land ratio	ecological area per capita
Total amount of regional ecological footprint	0.4632	0.5570	0.5744	0.5839	0.5406	0.5489	0.6545
Total amount of biocapacity	0.4833	0.5617	0.5965	0.6083	0.5822	0.5576	0.6646

### 3.2 Dynamic changes of biocapacity

**Fig 4:**
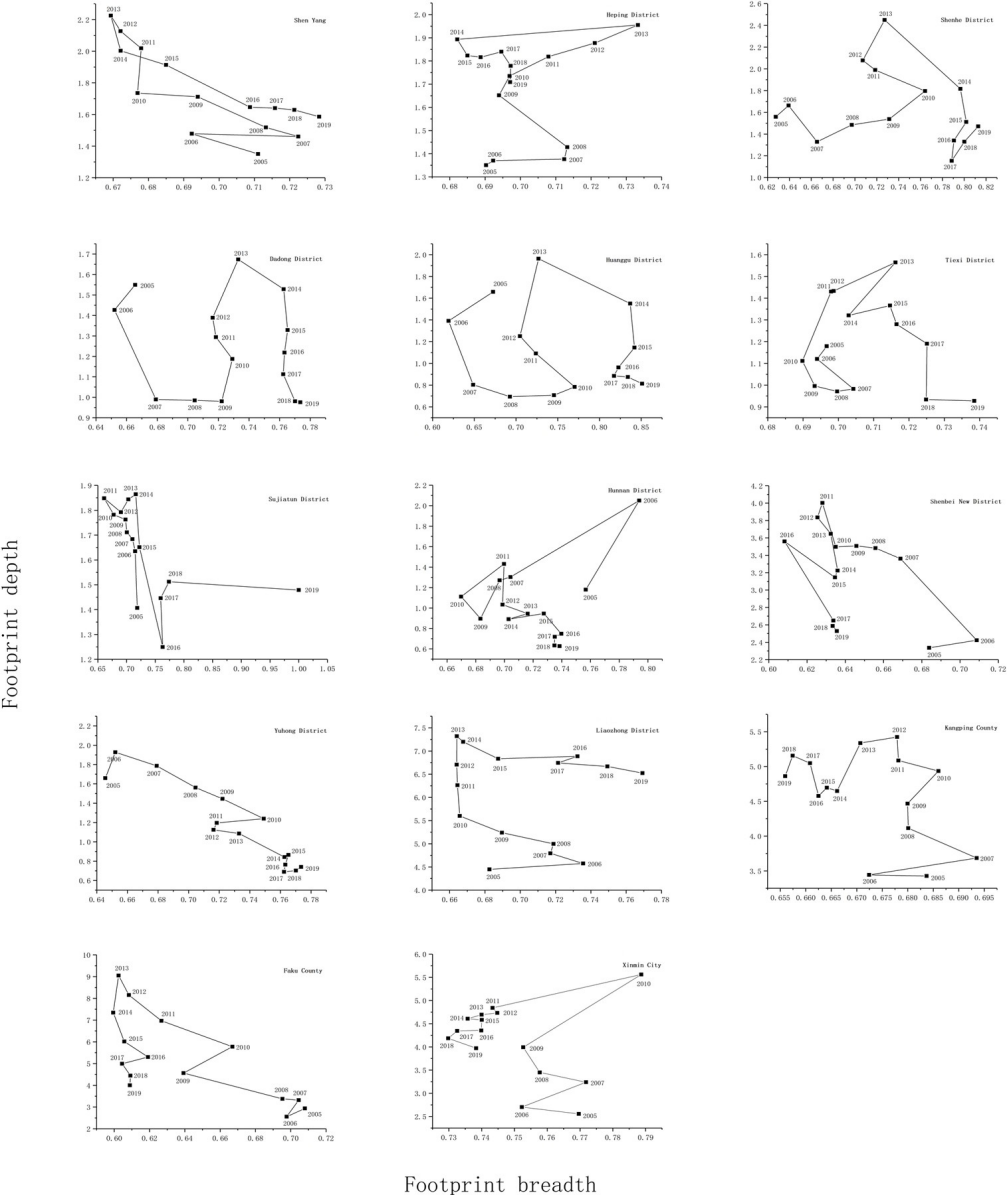
Time series of the relationship between footprint breadth and footprint depth in Shenyang (2005–2019).

Essentially influenced by the natural endowment and regional development orientation, the various occupied natural resources exhibited spatial distinctions in different regions of Shenyang. To better comprehend the relationship between flow capital occupation and stock capital consumption, time series analysis was adopted with the size of the ecological footprint as the x-coordinate and the depth of the footprint as the y-coordinate. Based on Fig 4, the dynamic variations of biocapacity in Shenyang can be classified into the following four types: (1) Closed type. Due to the sudden changes in the relative trend of footprint size and depth, certain closed parts were identified on the curve, especially in Shenyang City, Heping District, and Hunnan District. The size of ecological footprint has remained stable within the range of 0.67–0.73,the occupation of flow capital within the area was relatively stable, the depth of ecological footprint was greater than 1 mostly. It indicates that residents’ production and life not only consume the local flow ecological resources, but also continue to occupy the stock ecological resources. However, the depth of ecological footprint has declined after 2013, and the ecological pattern has been optimized. (2) Inverted U-type. With the gradual increase of footprint size, the footprint depth portrayed an up-to-down trend in general, especially in Shenhe District, Tiexi District, Yuhong District, Kangping County, Faku County, and Xinmin City. Under this type, the depth of ecological footprint of the municipal districts presented a changing trend of U- type with the increase of the size of ecological footprint. The consumption of ecological resources in this type of district has a tendency to shift from stock to flow, but the unfair phenomenon of regional flow capital occupation is becoming more and more obvious. In contrast, the occupation of flow capital tends to be fair in surrounding districts and counties. (3) S-type. With the gradual increase of footprint size, the footprint depth kept fluctuating, especially in Dadong District, Huanggu District, and Liaozhong district. The unfairness of flow capital occupation is increasingly prominent in these areas, ecological resource consumption is mainly based on "flow and stock". The unreasonable use of ecological resources is prominent, and adjustments and optimizations are urgently needed. (4) M-type. With the change of footprint size, footprint depth illustrated two prominent peaks, especially in Sujiatun District and Shenbei New District. The size of ecological footprint had a small change in these areas, the change in flow occupancy within the region was small with two peaks, indicating that the ecological resource consumption was over-utilizing the stock capital during the study period.Reasonable control measures should be taken to improve the utilization efficiency of ecological resources and reduce the waste of resources in the future development process.

Further meticulous analysis of the footprint depth of Shenyang revealed that it exhibited an up-to-down trend, and attained its peak in 2013. The upward part suggests that the capital stock must have been consumed exhaustively in each region and the resource constraints were intensified; the downward part suggests that the excessive consumption of regional resources had improved, and the urban ecological environment had been protected. Amongst them, significant regional differences were noticed regarding the depth of ecological footprint per capita, with Faku County being the upper bound and Hunnan District being the lower bound. This could be attributed to the rapid urbanization of surrounding counties/towns as well as the increased dependence of economic scale expansion on natural resources, which resulted in the gradual increase of ecological footprint per capita. The size of ecological footprint was noticed to be low with mild changes in general. Amongst them, Shenyang City, Heping District, Hunnan District, and Liaozhong District were under the evident influence of ecological footprint and biocapacity, and the size of footprint per capita illustrated significant fluctuations.

On the whole, since the 18th National Congress of the Communist Party of China, The construction of ecological civilization is gradually advancing in downtown areas and municipal districts of Shenyang, the excessive consumption of ecological resources has been alleviated, and the utilization of ecological resources is in a relatively ideal state of sustainable use, which should be maintained in the future development.With the rapid progress of urbanization in surrounding counties and towns, the expansion of economic scale is strongly dependent on natural resources, the irrational utilization of ecological resources has become more prominent, and the consumption of ecological resources is serious.Therefore, surrounding counties/towns have become key areas that need urgent attention and protection.

### 3.3 Multi-scale differentiation evaluation of biocapacity

**Fig 5:**
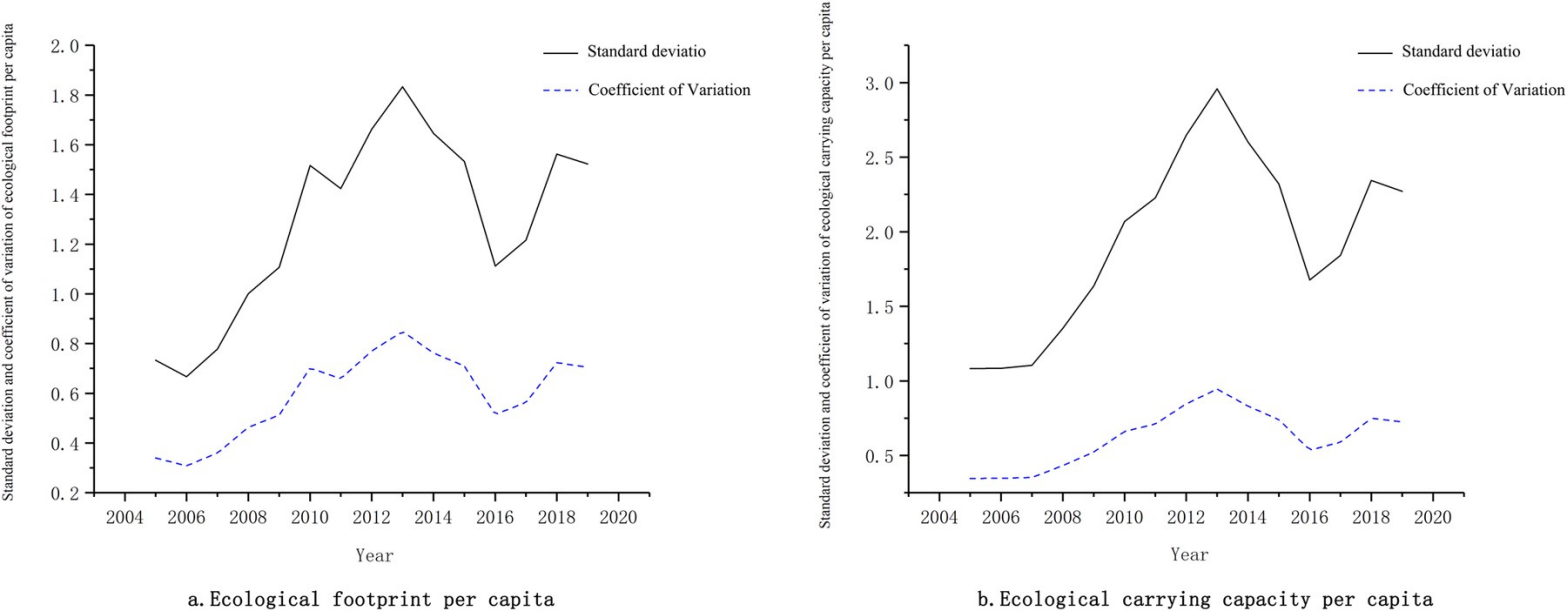
Ecological footprint per capita, standard deviation of ecological carrying capacity per capita and coefficient of variation in Shenyang. a. Ecological footprint per capita. b. Ecological carrying capacity per capita.

Owing to the distinctive natural conditions and urban development levels of 13 districts and counties in Shenyang, the spatial difference in the biocapacity of this region has for a long time existed on two scales and exhibited an evident scale effect (Fig 5). The standard deviation and variation coefficient of ecological footprint per capita and biocapacity per capita experienced a rapid rise, a sharp decline, and subsequently retained balance with mild fluctuations, with the standard deviation increasing by 0.86 and 1.32 and the variation coefficient increasing by 0.4 and 0.42, respectively. The differences of standard deviation generally tended to be higher than those of the variation coefficient. It indicates that the difference in biocapacity among districts and counties continued to increase during the study period. The primary reason was that with the rapid development of Shenyang’s regional economy, the polarization effect of administrative regions kept on escalating as well. Simultaneously, the constant adjustment and alteration of relevant policies, such as the trickle-down effect of urban economic growth, population spatial migration, and regional coordinated development, caused the resource utilization level among the districts and counties to vary dynamically, resulting in the persistent expansion of absolute differences and the fluctuations of relative differences.

**Fig 6:**
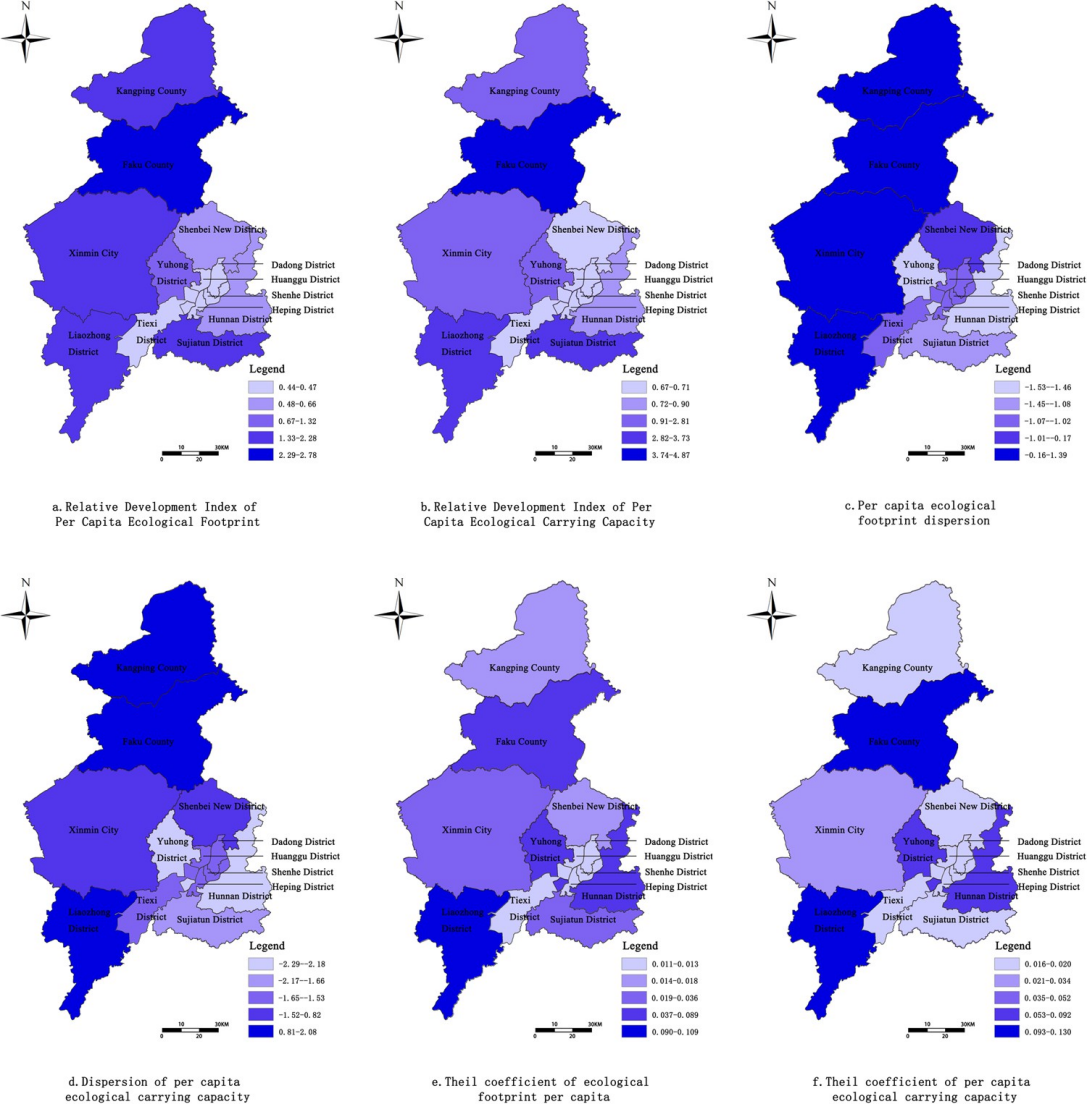
Ecological footprint per capita, relative development index of ecological carrying capacity per capita, deviation and Sill coefficient in Shenyang (2005–2019) (Image Source: https://www.naturalearthdata.com/downloads/10m-cultural-vectors/). a. Relative Development Index of Per Capita Ecological Footprint. b. Relative Development Index of Per Capita Ecological Carrying Capacity. c. Per capita ecological footprint dispersion. d. Dispersion of per capita ecological carrying capacity. e. Theil coefficient of ecological footprint per capita. f. Theil coefficient of per capita ecological carrying capacity.

The evaluation of relative development level of the change of biocapacity and its deviation from the average value of biocapacity of administrative regions (Fig 6) revealed the existence of significant spatial imbalance, demonstrating a distribution pattern of low in urban areas and high in surrounding areas. Based on the changing trend of relative development level and deviation, the gap between Faku County and urban areas was recorded to be the largest.,which were 2.78, 4.87and1.16, 2.08.Hunnan District had the smallest deviations in ecological footprint per capita and biocapacity per capita, which were -1.53 and -2.29, respectively.

Based on the spatial pattern of the Theil index (Fig 6), = Liaozhong District had the biggest Theil indexes, which were 0.109 and 0.130, respectively, and Shenhe District possessed the smallest Theil indexes, which were 0.109 and 0.130, respectively. Based on the cumulative analysis of the dynamic changes and spatial differences of biocapacity, it is found that the biocapacity in Liaozing District is the worst at this stage. In the process of rapid economic and social development and resource utilization, the natural capital flow is difficult to meet the regional consumption of resources, the natural capital stock is rapidly decreasing, and the environmental carrying and ecological protection pressure is huge. In the future development and construction, the level of economical and intensive use of land should be improved, and the transformation of sustainable utilization of ecological resources should be promoted. This indicates that on one hand, Shenyang responded to national policies quite actively, was committed to the coordinated development of economy and environment, adjusted its industrial structure, and improved the biocapacity of its downtown areas; on the other hand, with the rapid economic and social development of counties, regional resources were excessively exhausted, urban environment and ecosystem were not effectively protected, and the districts/counties with relatively backward economic status suffered from poor biocapacity.

## 4. Discussion

With the unceasing intensification of human social and economic activities in urban space and the constant increase of development intensity, the conflict between urban resources, environment, ecological utilization, and environmental protection has been gravely constraining high quality development of cities. At the same time, due to the spatial differences in the distribution of natural resources among cities in China, the development intensity and resource utilization tend to be different at different levels in various cities, while the biocapacity is restricted by regional scale and temporal and spatial conditions. Currently, majority of the existing studies on biocapacity primarily emphasize on large and medium scales, such as watershed [27], provincial-scale [28], and municipal-scale [29], while only a fewer biocapacity studies are available based on small and medium scales perspective. In this context, this paper describes the temporal and spatial changes of the biocapacity of Shenyang more intuitively from a multi-scale perspective, divides the types of regional changes, identifies the excessive use of flow capital in S-type and M-type, it’s pointed out that the allocation of ecological resources needs to be managed, controlled and protected, so as to promote regional coordinated development, which is innovative in research perspectives.

Studies concerning urban biocapacity focus more on the interactive relationship and internal mechanism between the urban socio-economic system and natural ecosystem. Such studies chiefly involve quantitative calculation [30] and temporal change [31] of regional biocapacity, and hardly investigate the evolution process of biocapacity from the perspective of regional difference and spatial differentiation. The process of rapid urbanization is bound to be accompanied with drastic land use changes, as a result, the biocapacity of Shenyang will also undergo tremendous changes in the spatial distribution, with evident scale effect and nonlinear characteristics.This paper adopts the ecological footprint spatio-temporal difference model and the multi-scale difference evaluation method, carries out multi-scale comparative study on the biocapacity at the municipal and district (county) level, identifies local variation feature information. It is determined that the biocapacity gap between Faku County and the urban area is the largest, and urgent attention and optimization are needed. These have made a breakthrough in the research content.

In the meantime, it should be understood that this paper is still constrained by the following conditions: (1) Considering the availability of data, the social statistical data obtained in this research may tend to be slightly different from the real conditions due to the varying statistical capacities; (2) The ground resolution of the satellite remote sensing image used in this paper is not completely unified, and slight deviation may be noticed between the statistical information of land boundaries and the real-time conditions.

## 5. Conclusion

Based on the panel data of Shenyang and its 13 districts and counties from 2005 to 2019, the temporal and spatial differentiation characteristics of urban biocapacity were analyzed, and the following conclusions were derived:

1. Municipal scale: The changes in ecological footprint and biocapacity of Shenyang conformed to the Kuznets cycle, and demonstrated an up-down-up trend. Affected by the urbanization process, the consumption of ecological resources in Shenyang reached its peak in 2013. The emergence of the point of inflexion coincides with the concept of the construction of ecological civilization. At the same time, People’s Government of Shenyang Municipality has passed a series of policy guidance including Environmental Protection Target Responsibility System Implementation Measures of Shenyang, Wetland Protection and Restoration Work Plan of Shenyang, Soil Pollution Prevention and Control Work Plan of Shenyang to strictly protect and control urban ecological natural resources, which positively affected the adjustment of the ecological pattern of Shenyang.County Scale: The spatial difference of biocapacity was quite Significant. Influenced by the regional economic development and industrial structure, the biocapacity of downtown areas and other surrounding districts and counties exhibited a two-level ascending hierarchical structure (from low to high). In the process of rapid urbanization, the ecological area per capita has decreased, which directly affects the level of regional biocapacity.
2. Through the evaluation of the time series of the relationship between footprint size and footprint depth in Shenyang, four major types were identified in its dynamic changes—closed type, inverted U-type, S-type, and M-type. And regional development of S-type and M-type consumes more flow capital.
3. The natural conditions and urban development level naturally tend to be different in every district and county of Shenyang. The spatial difference of biocapacity has for a long time existed on two scales, and established an evident scale effect. Superimposed on the dynamic change types and differences analysis, it is found that the biocapacity of Liaozhong District urgently needs attention and protection, and the need to improve the background ecological environment and enhance biocapacity is the most urgent.
